# Provisional Crown Dislodgement during Scuba Diving: A Case of Barotrauma

**DOI:** 10.1155/2013/749142

**Published:** 2013-07-28

**Authors:** Meenal Nitin Gulve, Nitin Dilip Gulve

**Affiliations:** ^1^Department of Conservative Dentistry and Endodontics, M.G.V.'s K.B.H. Dental College and Hospital, Mumbai Agra Road, Panchavati, Nashik 422002, India; ^2^Department of Orthodontics, Dentofacial Orthopedics and Dental Materials, M.G.V.'s K.B.H. Dental College and Hospital, Nashik 422002, India

## Abstract

Changes in ambient pressure, for example, during flying, diving, or hyperbaric oxygen therapy, can lead to barotrauma. Although it may seem that this issue was neglected in dental education and research in recent decades, familiarity with and understanding of these facts may be of importance for dental practitioners. We report the case of a patient who experienced barotrauma involving dislodgement of a provisional crown during scuba diving. Patients who are exposed to pressure changes as a part of their jobs or hobbies and their dentists should know the causes of barotrauma. In addition, the clinician must be aware of the possible influence of pressure changes on the retention of dental components.

## 1. Introduction

 In recent years it has become increasingly common to go to a tropical destination for a holiday [1]. There is often an opportunity to dive. Diving with self-contained underwater breathing apparatus (SCUBA) has witnessed explosive growth in the past decade, as 8.5 million people are certified in the United States alone. Around three million Europeans are thought to be recreational scuba divers, diving to depths up to around 40 meters sea water (msw). Additionally, there are approximately 800 professional civilians and 700 military divers registered in Germany as well as some 500 compressed air (caisson) workers, for example, for tunnel or bridge construction work [2]. With the increasing number of professional and leisure divers, the dentist may encounter related oral conditions that require immediate treatment. Although rare, dental emergencies while diving have been recognized as a potential cause of a diver suddenly becoming incapacitated, jeopardizing the safety of the affected person as well as others [3]. It is inevitable that the dental practitioner will have patients who participate in diving and they should be aware of a number of problems that a diver can experience that are associated with the teeth and related structures.

 An oral (dental or nondental) pain caused by change in barometric pressure in an otherwise asymptomatic organ is known as barodontalgia. The name of this dental pain was given the prefix “aero” (i.e., aerodontalgia) and was reported for the first time as an in-flight physiologic and pathologic phenomenon at the beginning of the 20th century. In the 1940s, with the appearance of SCUBA, many in-flight manifestations caused by barometric changes were found to be associated with diving as well. Consequently, the prefix was changed to “Baro” [4, 5]. Barodontalgia has been experienced on one or more occasions by 9.2% to 21.6% of American and Australian civilian scuba divers. Among military divers, an incidence of 17.3% was reported [6]. Barotrauma is defined as pressure induced damage that can occur both in high and low pressure [4]. Dental barotrauma can manifest itself as tooth fracture [7], restoration fracture, and dislodgement of the restoration [7, 8].

 In this paper, we present a case in which a patient experienced barotrauma involving a provisional crown while diving.

## 2. Diagnosis and Treatment

**Figure 1:**
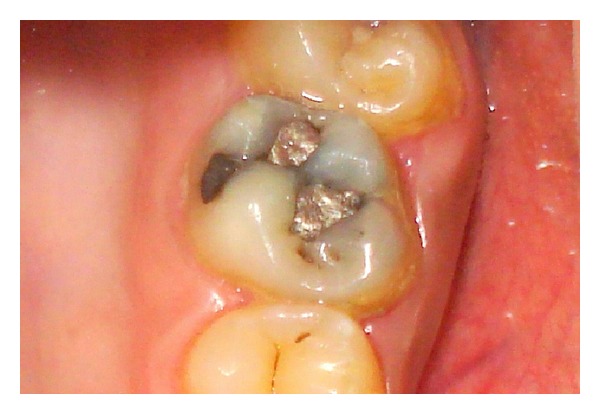
Amalgam restoration with secondary caries in the maxillary left first molar.

**Figure 2:**
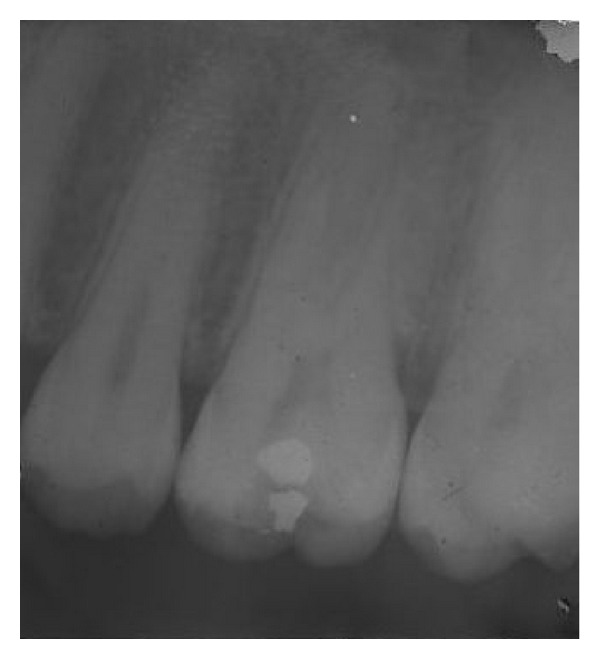
Radiolucent area approaching the pulp space with periodontal ligament space widening.

 A 25-year-old man presented with a chief complaint of spontaneous pain on the left side of his face for the past 3 days. History revealed intermittent pain to hot and cold stimuli for the past 1 month. The medical history was noncontributory. Clinical examination revealed an amalgam restoration with secondary caries in the maxillary left first molar (Figure 1). The tooth was tender to vertical percussion. Tooth mobility was within physiologic limit, and the gingival attachment apparatus was normal. Vitality testing of the involved tooth with heated gutta-percha (Dentsply Maillefer, Ballaigues, Switzerland) and dry ice (RC Ice; Prime Dental Product, Mumbai, India) caused an intense lingering pain, whereas electric pulp stimulation (Parkel Electronics Division, Farmingdale, NY, USA) caused a premature response. The preoperative radiographic evaluation showed evidence of radiolucent area in relation to maxillary left first molar approaching the pulp space with periodontal ligament space widening in relation to the mesial root (Figure 2). A diagnosis of symptomatic irreversible pulpitis with symptomatic apical periodontitis was made, and endodontic treatment was suggested to the patient.

**Figure 3:**
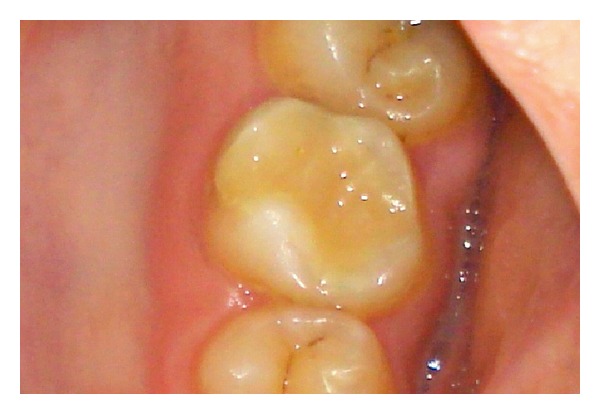
The tooth was restored with a posterior composite resin.

 Informed consent was taken. The tooth was anesthetized by using 1.8 mL 2% lignocaine containing 1 : 200,000 epinephrine. Endodontic treatment was completed and the tooth was then restored with a posterior composite resin core (Z350; 3M ESPE Dental Products, St. Paul, MN, USA) (Figure 3).

**Figure 4:**
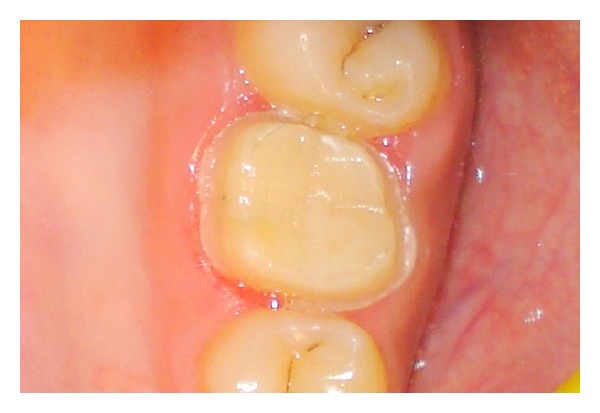
The tooth was prepared to receive metal-ceramic crown.

**Figure 5:**
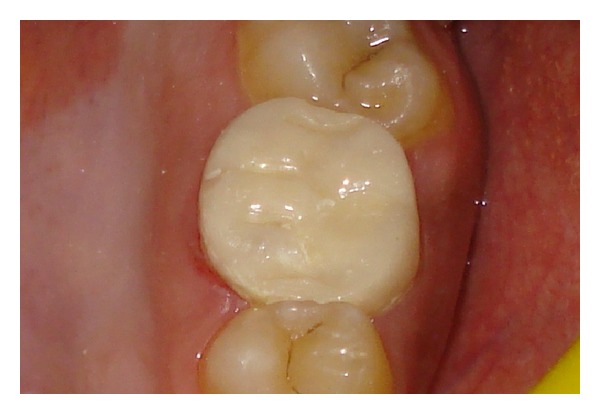
Provisional crown cemented with zinc phosphate cement.

 The tooth was prepared 1 week after endodontic treatment to receive a metal-ceramic crown in a conventional manner with the aim of obtaining a 6-degree convergence between walls (Figure 4). Gingival displacement was done, and two impressions were made: an alginate impression to prepare a provisional crown and another with hydrophilic polyvinyl siloxane impression material (Virtual; Ivoclar Vivadent, Schaan, Liechtenstein) to prepare metal-ceramic crown, poured using type IV dental stone (Kalrock; Kalabhai, India). The shade was determined with a shade guide. The patient was given a provisional crown, which was made from a bis-acryl material (Integrity; Dentsply Caulk, Konstanz, Germany) and was cemented with zinc phosphate cement (DeTrey Zinc; Dentsply DeTrey GmbH, Konstanz, Germany) (Figure 5). Excess cement was removed with a scalpel. The patient was recalled after 7 days to receive the final crown.

**Figure 6:**
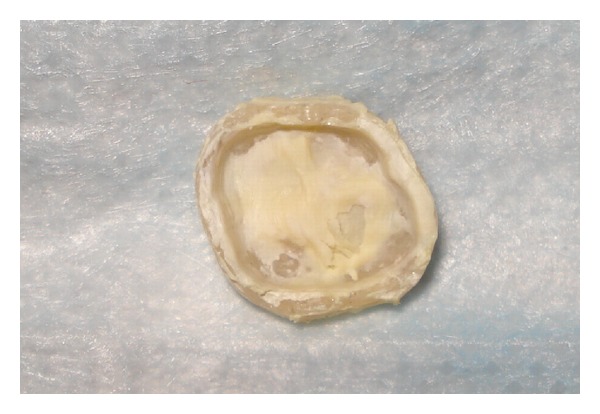
Dislodged provisional crown.

 However, the patient presented with dislodged provisional crown (Figure 6). He gave a history of SCUBA diving at a destination where he went for previously unplanned vacation. He reported that the provisional crown came out while he was diving at about 27 to 35 meters deep. After the crown dislodged, he had to keep it under his tongue until he returned to the surface to avoid aspiration or swallowing.

**Figure 7:**
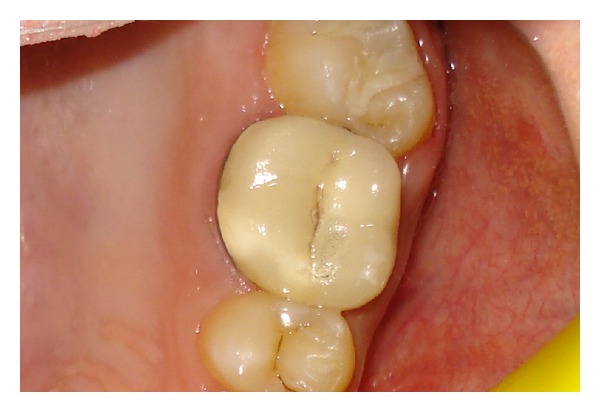
The metal-ceramic crown was cemented with self-adhesive resin cement.

 The tooth was cleaned of the temporary cement with a slurry of fine flour of pumice. The metal-ceramic crown was tried in to assess the marginal fit and contacts. The patient previewed and approved the shape and the shade of the crowns. The crown was cemented with self-adhesive resin cement (Multilink Speed; Ivoclar Vivadent AG, Schaan, Liechtenstein) in accordance with the manufacturer's instructions (Figure 7). Postoperative care instructions were given to the patient and recall appointments were scheduled.

## 3. Discussion

 In recent years it has become increasingly common to go to a tropical destination for a holiday. There is often an opportunity to dive. Also scuba diving is one of the fastest growing sports in the world [1]. In the case we presented, a leisure diver experienced barotrauma in which a provisional crown was dislodged while he was diving at about 27 to 35 meters deep under the surface of the sea. A diver at 30 m is subjected to four times the pressure encountered on the surface [9]. Although the exact mechanisms of barodontalgia and barotrauma are not known, the air trapped beneath a restoration or an endodontically treated tooth may be a factor [10].

 The possible reason for dislodging of provisional crown cemented with zinc phosphate could be associated Boyle's law, which states that, at a constant temperature, the volume of a gas varies inversely with the surrounding pressure. As pressure increases, the volume of a confined gas decreases. Vice versa, volume increases as pressure decreases [9]. The problem arises when the enclosed spaces containing gases cannot expand or contract to adjust the internal pressure to correspond to the outer pressure. During the mixing process of luting cement, air may become incorporated into the mixture, forming voids [11]. The expansion or contraction of these microbubbles during pressure cycling, which eventually led to disruption and weakening of cement layer, could affect the retention [12]. Davidson et al. [13] found that microcracks appear as a result of volumetric contraction in luting cements and, when subjected to the pressure cycling, may have produced tensile stresses that exceeded the cohesive and adhesive strength of the material, resulting in the significant reduction in tensile bond strength. 

 Lyons et al. [12] studied the effect of cycling environmental pressure changes on the retention of crowns on the extracted teeth. The crowns that were cemented with either zinc phosphate or glass ionomer cement had significantly reduced retention (in approximately 90% and 50%, resp.), whereas crowns that were cemented with resin cement were not subjected to reduced retention after pressure cycling. Moreover, microleakage was detected in the zinc phosphate and glass ionomer cements after pressure cycling, whereas no microleakage was detected in the resin cement. Musajo et al. [9] also reported similar results with crowns cemented with zinc phosphate cement. Gulve et. al [14] found that the pullout strength of orthodontic bands cemented with glass ionomer cement is reduced after pressure cycling.

 It seems that, currently, the incidence of in-flight dental barotrauma is relatively low compared to reported incidences from the first half of the 20th century owing to the current inside compression of airplane chambers. The pressure inside the chamber fits pressure at altitudes of 5,000 to 10,000 feet, whereas cases of dental barotraumas were reported in pressure at altitudes of 18,000 feet and higher [15]. However, whereas in flight the theoretically possible pressure changes range from 1 atm (at ground level) to 0 atm (at outer space), in diving the changes are more significant, since each descent of 10 meters (32.8 feet) elevates the pressure by 1 atm. Thus, the barometric changes during diving may be more significant and responsible for more frequent and/or severe related pathogenesis than in-flight barometric changes. However, while aviators are obligated to be examined periodically, it is a rarity among divers [16].

 Divers and aircrew medical examiners should recommend their aviator and diver patients to be periodically examined by a dental practitioner who is familiar with the subject. It is important for a dentist to be aware of the effect of pressure changes on dental components in terms of retentive strength, as danger resulting from dislodgement of component during a dive is obvious.

The dentist should advise patients not to dive while having a provisional crown or temporary cement in the mouth.
